# Deciphering the omicron variant: integrated omics analysis reveals critical biomarkers and pathophysiological pathways

**DOI:** 10.1186/s12967-024-05022-z

**Published:** 2024-02-29

**Authors:** Qianyue Yang, Zhiwei Lin, Mingshan Xue, Yueting Jiang, Libing Chen, Jiahong Chen, Yuhong Liao, Jiali Lv, Baojun Guo, Peiyan Zheng, Huimin Huang, Baoqing Sun

**Affiliations:** 1 Department of Clinical Laboratory, National Center for Respiratory Medicine, National Clinical Research Center for Respiratory Disease, State Key Laboratory of Respiratory Disease, Guangzhou Institute of Respiratory Health, The First Affiliated Hospital of Guangzhou Medical University, Guangzhou, 510120 Guangdong China; 2 Respiratory Mechanics Laboratory, National Center for Respiratory Medicine, Guangzhou Institute of Respiratory Health, First Affiliated Hospital of Guangzhou Medical University, Guangzhou, 510120 Guangdong China; 3 Guangzhou Laboratory, Guangzhou International Bio Island, XingDaoHuanBei Road, Guangzhou, 510005 Guangdong Province China

**Keywords:** Omicron variant, SARS-CoV-2, Bioinformatics, Coagulation, Hemostasis

## Abstract

**Background:**

The rapid emergence and global dissemination of the Omicron variant of SARS-CoV-2 have posed formidable challenges in public health. This scenario underscores the urgent need for an enhanced understanding of Omicron's pathophysiological mechanisms to guide clinical management and shape public health strategies. Our study is aimed at deciphering the intricate molecular mechanisms underlying Omicron infections, particularly focusing on the identification of specific biomarkers.

**Methods:**

This investigation employed a robust and systematic approach, initially encompassing 15 Omicron-infected patients and an equal number of healthy controls, followed by a validation cohort of 20 individuals per group. The study's methodological framework included a comprehensive multi-omics analysis that integrated proteomics and metabolomics, augmented by extensive bioinformatics. Proteomic exploration was conducted via an advanced Ultra-High-Performance Liquid Chromatography (UHPLC) system linked with mass spectrometry. Concurrently, metabolomic profiling was executed using an Ultra-Performance Liquid Chromatography (UPLC) system. The bioinformatics component, fundamental to this research, entailed an exhaustive analysis of protein–protein interactions, pathway enrichment, and metabolic network dynamics, utilizing state-of-the-art tools such as the STRING database and Cytoscape software, ensuring a holistic interpretation of the data.

**Results:**

Our proteomic inquiry identified eight notably dysregulated proteins (THBS1, ACTN1, ACTC1, POTEF, ACTB, TPM4, VCL, ICAM1) in individuals infected with the Omicron variant. These proteins play critical roles in essential physiological processes, especially within the coagulation cascade and hemostatic mechanisms, suggesting their significant involvement in the pathogenesis of Omicron infection. Complementing these proteomic insights, metabolomic analysis discerned 146 differentially expressed metabolites, intricately associated with pivotal metabolic pathways such as tryptophan metabolism, retinol metabolism, and steroid hormone biosynthesis. This comprehensive metabolic profiling sheds light on the systemic implications of Omicron infection, underscoring profound alterations in metabolic equilibrium.

**Conclusions:**

This study substantially enriches our comprehension of the physiological ramifications induced by the Omicron variant, with a particular emphasis on the pivotal roles of coagulation and platelet pathways in disease pathogenesis. The discovery of these specific biomarkers illuminates their potential as critical targets for diagnostic and therapeutic strategies, providing invaluable insights for the development of tailored treatments and enhancing patient care in the dynamic context of the ongoing pandemic.

**Supplementary Information:**

The online version contains supplementary material available at 10.1186/s12967-024-05022-z.

## Introduction

In November 2021, amidst the ongoing global pandemic of the novel coronavirus (SARS-CoV-2), South African scientists identified a new variant of concern (VOC), Omicron, which was promptly classified as such by the World Health Organization (WHO) on November 26, 2021 [1]. Despite increasing vaccination rates, Omicron and its subvariants continue to spread rapidly worldwide [2]. Compared to the early wild-type virus, Omicron exhibits a higher transmissibility and immune evasion capability, posing a significant public health challenge [3]. Studies have shown that both Omicron and the Delta variant can evade host immune responses through single-point mutations on the spike protein [4], with Omicron having a stronger binding affinity to receptors, thus enhancing its potential for immune evasion [5]. Although Omicron may be less pathogenic, it still poses a lethal threat to immunocompromised individuals or patients undergoing immunosuppressive therapy. The pathogenesis of Omicron is increasingly evidenced to be closely associated with cytokine storms [6], systemic inflammatory responses [6], and platelet count abnormalities [7]. The mechanisms related to platelet counts remain inadequately explained in the context of SARS-CoV-2 infection.

Platelets (PLT), key cellular components in maintaining vascular integrity and initiating hemostasis, are produced by megakaryocytes in the bone marrow and circulate in the blood as cell fragments [8]. They play a crucial role in the development, progression, and ultimate outcome of many diseases, intimately associated with platelet-mediated hemostasis and thrombosis, which may trigger adverse inflammatory responses [9]. Platelets can release serotonin and participate in immune responses by promoting T-cell activation, thus modulating immune reactions [10]. Their inherent adhesiveness allows them to interact with a variety of cells from both the innate and adaptive immune systems [11]. Studies, including that by Sevilya et al., have shown a direct link between platelet activation and SARS-CoV-2 infection [12], and it has been suggested that the expression of the angiotensin-converting enzyme 2 (ACE2) receptor and the transmembrane serine protease transmembrane serine protease 2 (TMPRSS2) on platelets and megakaryocytes play a role in mediating viral infection [13, 14]. Moreover, the functional heterogeneity of platelet aggregation is thought to play a role in the pathogenesis of Omicron infection, contributing to the regulation of immune function within the hematological ecosystem [15].

While current research on COVID-19 pathogenesis and the coagulation/hemostasis pathways are ongoing, most studies still focus on the perspective of biomarkers [16, 17], and there are few studies on Omicron patients. This study aims to delve into the molecular interactions among key differential proteins of the Omicron variant, to identify potential targets for intervention, and to further analyze the metabolomic characteristics in the peripheral blood of Omicron patients. The urgency of this research stems from the necessity to understand the intricate interplay between the virus's pathogenesis and the host's immune response. Given the global impact of Omicron, elucidating these mechanisms is not only of scientific interest but is imperative for the development of targeted therapies and for informing public health strategies.

## Methodology

### Biospecimen collection and clinical data acquisition

**Fig. 1:**
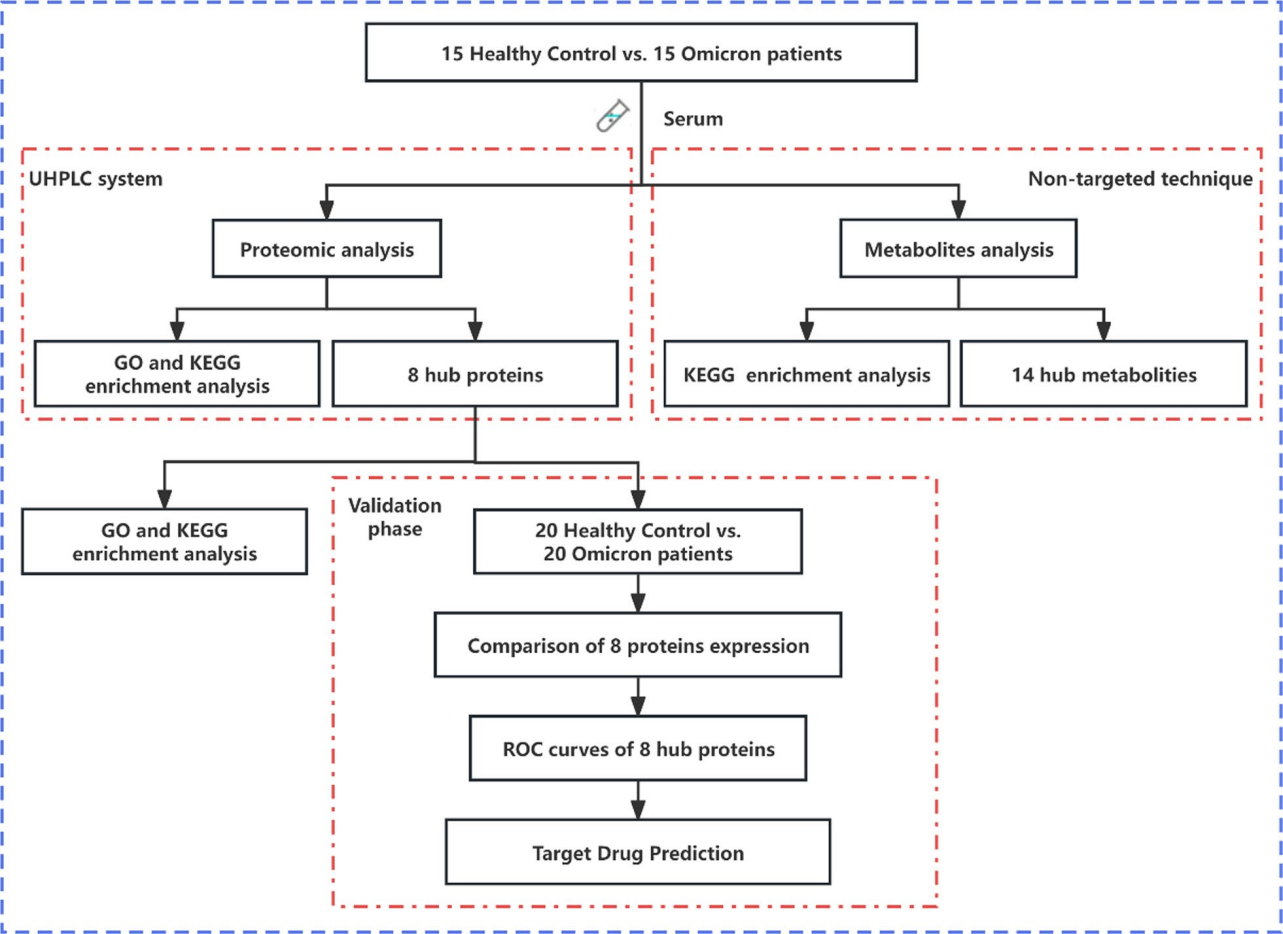
A visual representation of the study design. It is mainly divided into three parts: proteomics detection, metabolomics detection and validation queue detection. This structured approach ensures a thorough analysis of the biological samples, integrating proteomic and metabolomic data to validate the identified biomarkers and pathways relevant to the study's focus

The study systematically collected biospecimens and clinical data across two distinct phases: screening and validation, ensuring a robust analytical framework. In the screening phase, 15 patients diagnosed with Omicron variant infections via nucleic acid testing were recruited from the First Affiliated Hospital of Guangzhou Medical University. Diagnoses were established according to the latest COVID-19 Omicron diagnostic and treatment guidelines released by the World Health Organization, coupled with clinical symptoms and pharyngeal swab nucleic acid test results. Additionally, 15 healthy volunteers, matched by age and gender, were selected as controls. Comprehensive demographic, clinical, and laboratory data were compiled for both groups, followed by serum collection for in-depth proteomic and metabolomic analyses. The validation phase further substantiated our initial findings through an expanded cohort of 20 Omicron patients and 20 controls, employing identical proteomics methodologies and Receiver operating characteristic (ROC) analyses to ensure the reproducibility and validity of our screening phase outcomes. Informed consent was obtained from all participants, and the study protocol was approved by the Ethics Committee of the First Affiliated Hospital of Guangzhou Medical University (Ethical Approval Numbers: 202001134 and 202115202). A schematic representation of the study design can be found in Fig. 1.

### Proteomic analysis

Upon thawing, serum samples were treated with methanol/acetonitrile (1:1, volume ratio, Sigma-Aldrich, St. Louis, MO, USA) to achieve a total volume ratio of 2:2:1 (excluding water). The samples were vortexed on ice for 30 s, subjected to high-intensity ultrasonication for 10 min, and then left to stand at -20 °C for one hour. Subsequently, the samples were centrifuged at 4 °C (12,000g, 15 min), and the supernatant was collected and dried at room temperature. The dried samples were reconstituted in acetonitrile/water (1:1, volume ratio, Sigma-Aldrich, St. Louis, MO, USA), vortexed and ultrasonicated again, and centrifuged to remove any precipitate. The supernatant was stored at −80 °C until analysis. The samples were resolved in solvent A (0.1% formic acid in 2% acetonitrile/water, Sigma-Aldrich, St. Louis, MO, USA) and separated using a homemade reverse-phase liquid chromatography column (25 cm length, 75/100 μm inner diameter). Chromatographic separation was carried out on a nanoElute Ultra-High-Performance Liquid Chromatography (UHPLC) system (Bruker Daltonics, Billerica, MA, USA) at a flow rate of 450 nL/min, followed by electrospray ionization through a capillary source and mass spectrometric analysis using a timsTOF Pro (Bruker Daltonics, Billerica, MA, USA) in PASEF mode with a dynamic exclusion time of 30 s.

### Metabolomic analysis

Metabolite separation was conducted using a Waters ACQUITY UltraPerformance Liquid Chromatography (Waters ACQUITY UPLC) System (Waters Corporation, Milford, MA, USA) equipped with a BEH C18 column. Chromatographic conditions included a 10 µL injection volume, a flow rate of 400 µL/min, and a column temperature maintained at 40 °C. Metabolomics data were processed using MassLynx (Waters Corporation, Milford, MA, USA), which entailed peak extraction, alignment, and retention time correction, ensuring that the mass accuracy was controlled within 20 ppm to guarantee data accuracy.

### Bioinformatics analysis

Proteomics data were analyzed using the limma package in R software (version 3.6.3) to identify differentially expressed proteins (DEPs) with p < 0.05 and Log_2_FC > 1 [18, 19]. Heatmaps, volcano plots, and boxplots were generated using the "heatmap" and "ggplot2" packages in R (version 3.3.3). Proteins were annotated using the UniProt-GOA database (http://www.ebi.ac.uk/GOA), and enriched pathways were determined using Kyoto Encyclopedia of Genes and Genomes (KEGG) analysis. Protein–protein interaction networks (PPI) for the DEPs were constructed using the STRING database (https://string-db.org/), and further analysis of the PPI was carried out in Cytoscape software using cytohubba and MCODE plugins to identify and analyze key hub proteins, which were then subjected to GO/KEGG enrichment analysis.

Metabolomics data were normalized and integrated using support vector regression and analyzed for significant differences using orthogonal partial least squares-discriminant analysis (OPLS-DA). Variable importance in projection (VIP) values were calculated for each metabolite, with a VIP > 1 considered significant (P < 0.05) [20]. Pathway enrichment analysis for these differentially expressed metabolites was performed using GO/KEGG databases to identify significantly enriched pathways.

### Prediction of potential therapeutic agents

The DSigDB database (http://tanlab.ucdenver.edu/DSigDB) was utilized to predict potential therapeutic agents for COVID-19 based on protein–drug interaction data. The thresholds set for selection were False Discovery Rate (FDR) < 0.05 and composite score > 5000.

### Statistical analysis

Statistical analyses were conducted using SPSS software version 27.0, with continuous variables expressed as median and interquartile range (IQR). The Mann–Whitney U test (for two groups) and independent samples t-test were employed to assess differences in continuous variables. A P < 0.05 was considered statistically significant. Receiver operating characteristic (ROC) curves were plotted using Graphpad Prism software (version 9.5.1) to determine the sensitivity and specificity of hub genes. The area under the ROC curve (AUC) was measured, with an AUC > 0.7000 indicating diagnostic significance for the differentially expressed proteins.

## Results

### Clinical characteristics of the screening cohort

In terms of clinical parameters, compared to the healthy control group, the Omicron patient group exhibited elevated levels of Neutrophil percentage (NEU%), Monocyte count and percentage (MONO% and MONO), Basophils percentage (BASO%), International Normalized Ratio (INR), Fibrinogen (FIB), D-Dimer (D-D), C-reactive protein (CRP), Serum Amyloid A (SAA), and Procalcitonin (PCT) (P < 0.05). Conversely, there was a significant decrease in Lymphocyte count and percentage (LYM% and LYM), Eosinophils count and percentage (EOS and EOS%), Basophil count (BASO), and Prothrombin Time Activity (PTTA) (P < 0.05). These differences were statistically significant (Table 1).

**Table 1:** Basic information of healthy controls and omicron patients

	Healthy controls	Omicron	P
N	15	15	
Age	49.00 (37.00–61.00)	45.00 (29.00–57.00)	0.578
WBC, 10^9^/L	5.90 (4.80–6.60)	5.33 (4.12–7.17)	0.817
NEU %	58.20 (52.20–62.30)	67.90 (59.20–76.20)	0.024
LYM %	29.80 (27.80–37.60)	18.80 (13.00–30.10)	0.002
MONO %	6.60 (6.40–8.20)	10.10 (8.50–13.70)	0.001
EOS %	3.20 (1.50–4.00)	0.40 (0.30–1.10)	< 0.001
BASO %	0.13 (0.12–0.23)	0.42 (0.41–0.66)	< 0.001
NEU, 10^9^/L	3.40 (3.00–3.70)	3.71 (2.59–5.03)	0.380
LYM, 10^9^/L	1.80 (1.60–2.20)	1.30 (0.69–1.39)	< 0.001
MONO,10^9^/L	0.42 (0.40–0.51)	0.57 (0.42–0.87)	0.040
EOS, 10^9^/L	0.19 (0.16–0.22)	0.02 (0.01–0.06)	< 0.001
BASO, 10^9/L	0.07 (0.05–0.08)	0.03 (0.02–0.04)	< 0.001
PT, S	12.80 (12.50–13.50)	12.80 (11.30–15.30)	0.844
INR	0.60 (0.40–0.90)	0.98 (0.85–1.35)	< 0.001
PTTA, %	96.00 (87.00–106.00)	82.00 (73.00–89.00)	0.004
FIB, g/L	3.22 (2.45–3.26)	3.48 (3.31–3.65)	0.003
APTT, S	33.10 (32.10–36.20)	36.40 (32.20–41.20)	0.171
TT, S	16.20 (15.50–17.80)	17.80 (16.20–18.20)	0.224
D-Dimer, μg/L	333.00 (258.00–364.00)	486.00 (452.00–852.00)	< 0.001
CRP, mg/dL	2.50 (1.70–3.00)	5.30 (2.82–9.44)	0.005
SAA, mg/L	6.30 (2.90–8.40)	43.60 (29.40–67.60)	< 0.001
PCT, ng/mL	0.02 (0.01–0.04)	0.09 (0.05–0.14)	< 0.001

*WBC* White blood cell, *NEU* Neutrophil, *LYM* Lymphocyte, *MONO* Monocyte, *EOS* Eosinophils, *BASO* Basophil, *PT* Prothrombin time, *INR* International normalized ratio, *PTTA* Prothrombin activity, *FIB* Fibrinogen, *APTT* Activated partial thromboplastin time, *TT* Thrombin, *DD* D-Dimer, *CRP* C-reactive protein, *SAA* Serumamyloid A, *PCT* Procalcitonin

### Proteomic profile and functional alterations associated with omicron

**Fig. 2:**
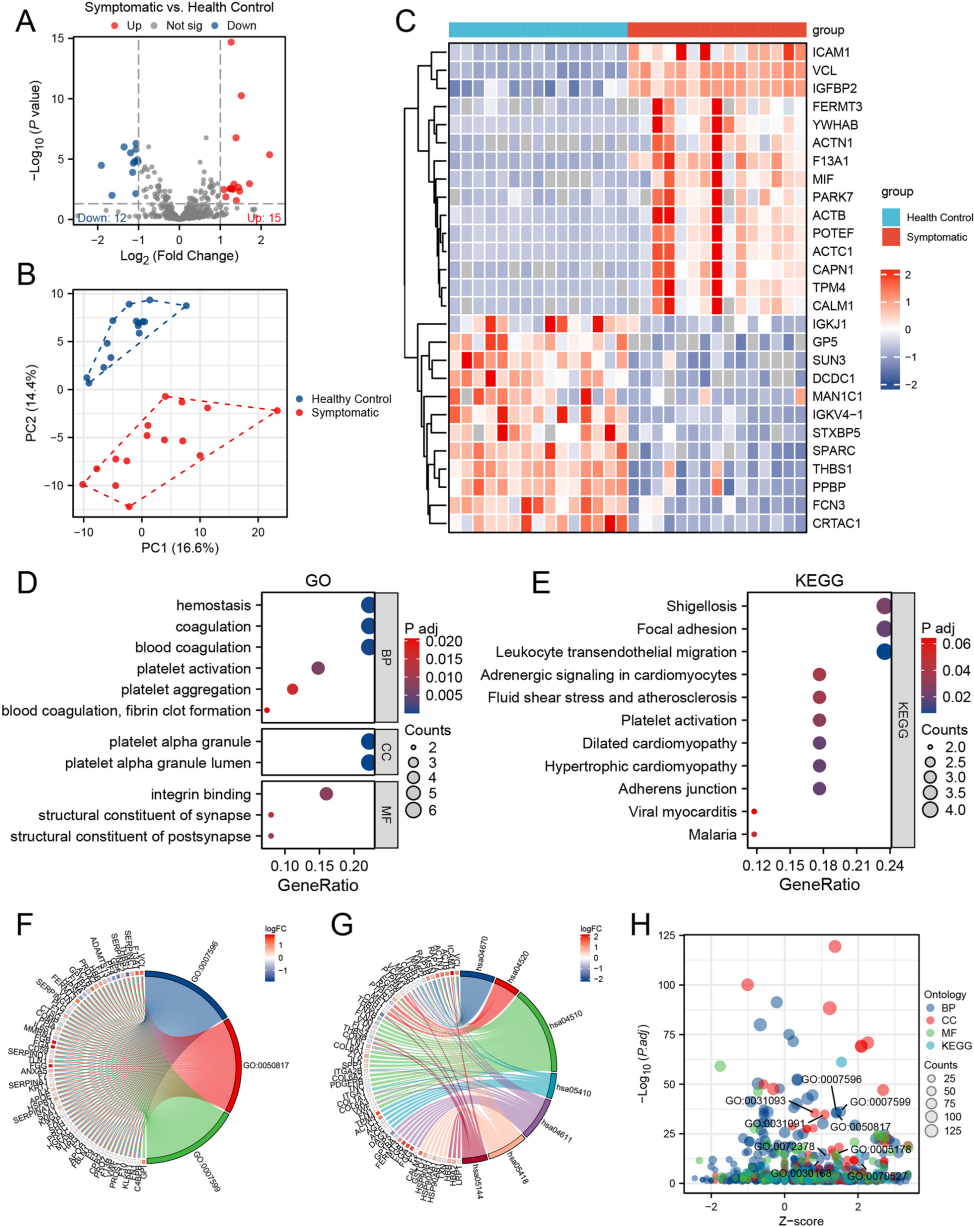
Analysis procedures for subject inclusion and overall analysis of bioinformation. **A** The volcano plots of differentially expressed proteins (DEPs); **B** Principal Component Analysis (PCA) of 2 Groups; **C** Heatmap of DEPs; **D**, **E** Gene Ontology (GO) and Kyoto Encyclopedia of Genes and Genomes (KEGG) analyses reveal the functional categories and pathways enriched among the 27 DEPs. The GO analysis primarily focuses on hemostasis, platelet alpha granule, and integrin binding pathways. The KEGG analysis emphasizes pathways associated with shigellosis, focal adhesion, and leukocyte transendothelial migration. **F**–**H** Chord diagrams illustrate the intricate relationships between GO/KEGG terms. *BP* Biological process, *CC* Cellular component, *MF* Molecular function, *PCA* principal component analysis

Using the UHPLC system, a total of 746 proteins were identified in the serum samples of both groups. Based on the criteria of | log2(FC) |> 1 and p-value < 0.05, a total of 27 differentially expressed proteins (DEPs) were identified, which included 15 upregulated proteins and 12 downregulated proteins, as shown in Fig. 2A (Additional file 1: Table S1). Principal Component Analysis (PCA), conducted using the normalized expression matrix, confirmed the variability between the proteomes of the samples (Fig. 2B). The heatmap of the aforementioned differentially expressed proteins is depicted in Fig. 2C.

GO and KEGG enrichment analyses were conducted on the differentially expressed proteins (Additional file 1: Table S2). These proteins were significantly enriched in GO pathways related to hemostasis, coagulation, and blood clotting, and were significantly enriched in the KEGG pathways related to Shigella and focal adhesion kinase pathways (Figs. 2D–H).

### Selection and functional enrichment analysis of omicron hub proteins

**Fig. 3:**
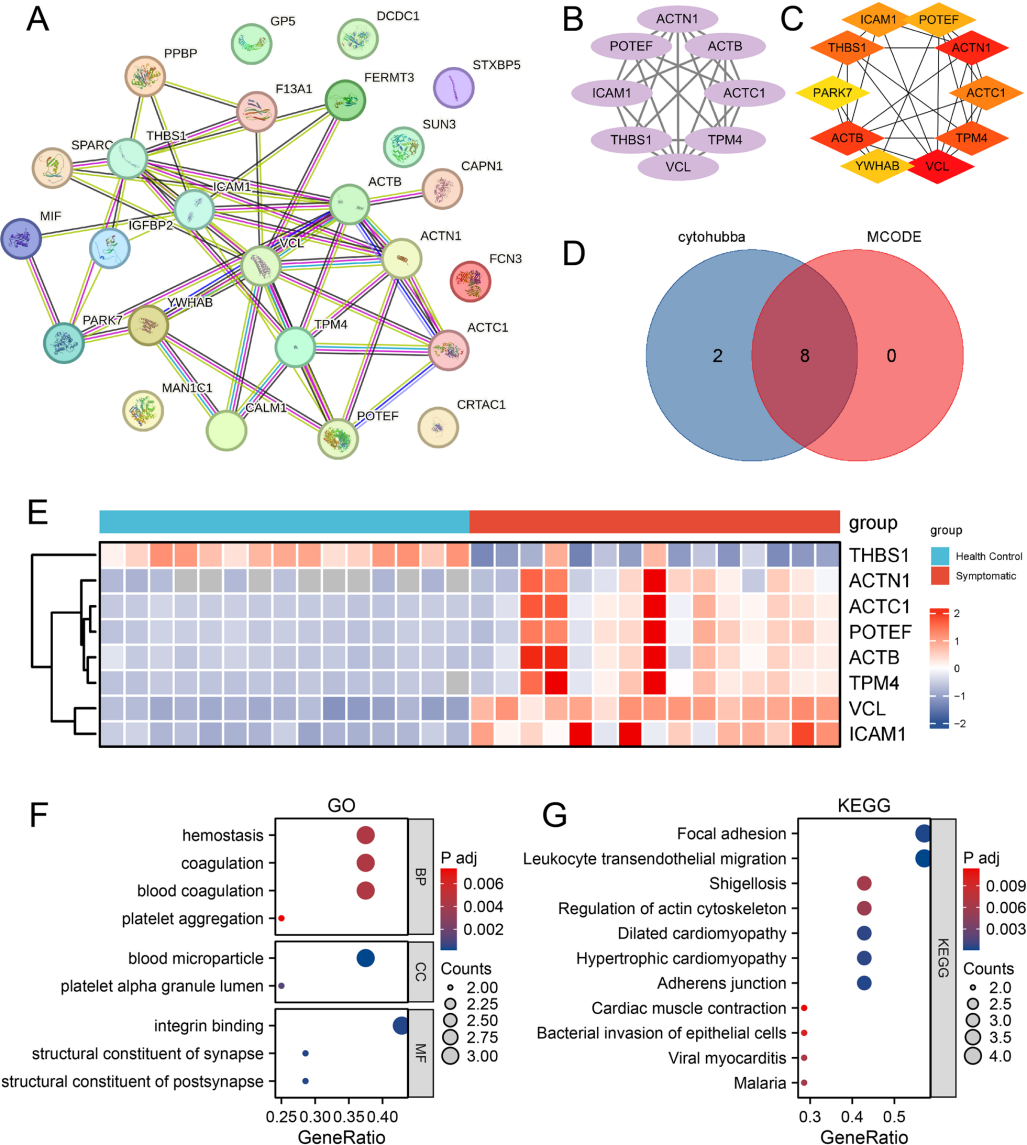
Secondary screening of functional pathway core protein sites. **A** STRING of 27 differentially expressed proteins (DEPs); **B** 8 MCODE hub genes:: THBS1, ACTN1, ACTC1, POTEF, ACTB, TPM4, VCL, and ICAM1; **C** 10 cytohubba-MCC hub genes: THBS1, ACTN1, ACTC1, POTEF, ACTB, TPM4, VCL, ICAM1,PARK7, and YWHAB; **D** VENN of MCODE hub genes and cytohubba-MCC hub genes: THBS1, ACTN1, ACTC1, POTEF, ACTB, TPM4, VCL, and ICAM1; **E** Heatmap of 8 hub proteins. **F**, **H** Gene Ontology (GO) analysis reveals enrichment in hemostasis, blood microparticle, and integrin binding pathways, while Kyoto Encyclopedia of Genes and Genomes (KEGG) pathways focus on focal adhesion, leukocyte transendothelial migration, and shigellosis

Protein–protein interaction networks were constructed using the STRING database (Fig. 3A), and core hub proteins for both groups were identified through the MCC algorithm of the cytohubba plugin in Cytoscape software (Fig. 3C), leading to the selection of 10 hub proteins: THBS1, ACTN1, ACTC1, POTEF, ACTB, TPM4, VCL, ICAM1, PARK7, and YWHAB. Further analysis confirmed 8 of these hub proteins by MCODE: THBS1, ACTN1, ACTC1, POTEF, ACTB, TPM4, VCL, and ICAM1 (Fig. 3B). Venn diagrams from both methods confirmed the 8 common differentially expressed hub proteins (Fig. 3D). Heatmap analysis revealed an upregulated trend in Omicron patients compared to the healthy control group for all hub proteins except THBS1 (Fig. 3E).

Enrichment analysis of the 8 differentially expressed hub proteins was performed using GO/KEGG pathways (Additional file 1: Table S3). The results showed that the most enriched GO pathways included hemostasis, coagulation, and platelet aggregation, while the most enriched KEGG pathways were focal adhesion kinase, leukocyte transendothelial migration, Shigella, and actin cytoskeleton among others (Figs. 3F, G).

### Clinical characteristics of the validation cohort

In the validation cohort, significant differences were observed when comparing Omicron patients (n = 20) to the healthy control group (n = 20). There was a notable increase in Neutrophil percentage (NEU%), Monocyte percentage (MONO%), Prothrombin time (PT), Fibrinogen (FIB), Activated partial thromboplastin time (APTT), D-Dimer (D-D), C-reactive protein (CRP), Serum amyloid (SAA), and Procalcitonin (PCT) levels (P < 0.05) in the Omicron patients. Conversely, a significant decrease was found in Lymphocyte percentage (LYM% and LYM), Eosinophils (EOS), Basophil percentage (BASO%), and Prothrombin Activity (PTTA) (P < 0.05). These differences were statistically significant (Table 2).

**Table 2:** Basic information of participants in the second batch verification queue

	Healthy controls	Omicron	P
N	20	20	
Age	54.00 (36.75–58.75)	53.00 (46.00–62.00)	0.580
WBC, 10^9^/L	6.64 (5.35–9.27)	5.80 (4.62–7.85)	0.229
NEU %	53.88 (44.30–61.44)	92.66 (73.34–105.28)	< 0.001
LYM %	29.37 (26.30–34.81)	16.71 (13.34–28.89)	< 0.001
MONO %	6.98 (5.34–8.29)	12.33 (10.61–14.60)	< 0.001
EOS %	1.45 (0.88–3.38)	1.65 (0.93–2.10)	0.839
BASO %	0.86 (0.38–1.13)	0.17 (0.10–0.22)	< 0.001
NEU, 10^9^/L	5.00 (3.67–6.05)	5.28 (2.65–5.53)	0.655
LYM, 10^9^/L	2.40 (1.80–3.15)	1.50 (0.80–2.30)	0.004
MONO, 10^9^/L	0.44 (0.26–0.65)	0.42 (0.37–0.66)	0.960
EOS, 10^9^/L	0.22 (0.16–0.37)	0.15 (0.08–0.21)	0.004
BASO, 10^9^/L	0.02 (0.01–0.03)	0.03 (0.02–0.03)	0.194
PT, S	12.45 (11.73–13.40)	14.00 (12.85–14.58)	0.003
INR	0.75 (0.60–0.90)	0.90 (0.80–1.18)	0.060
PTTA, %	97.00 (92.43–109.00)	83.20 (76.25–94.10)	< 0.001
FIB, g/L	2.90 (2.53–3.45)	4.74 (3.53–5.52)	< 0.001
APTT, S	35.70 (31.50–40.15)	41.40 (31.85–47.18)	0.048
TT, S	16.15 (15.23–18.78)	16.55 (15.20–18.33)	0.745
D-Dimer, μg/L	297.00 (211.00–364.75)	510.00 (439.50–603.50)	< 0.001
CRP, mg/dL	2.75 (1.65–4.13)	8.50 (5.80–10.40)	< 0.001
SAA, mg/L	5.50 (3.10–8.38)	35.75 (30.48–44.88)	< 0.001
PCT, ng/mL	0.02 (0.01–0.05)	0.09 (0.06–0.15)	< 0.001

*WBC* White blood cell, *NEU* Neutrophil, *LYM* Lymphocyte, *MONO* Monocyte, *EOS* Eosinophils, *BASO* Basophil, *PT* Prothrombin time, *INR* International normalized ratio, *PTTA* Prothrombin activity, *FIB* Fibrinogen, *APTT* Activated partial thromboplastin time, *TT* Thrombin, *DD* D-Dimer, *CRP* C-reactive protein, *SAA* Serumamyloid A, *PCT* Procalcitonin

### Validation of differential hub protein expression and diagnostic value

**Fig. 4:**
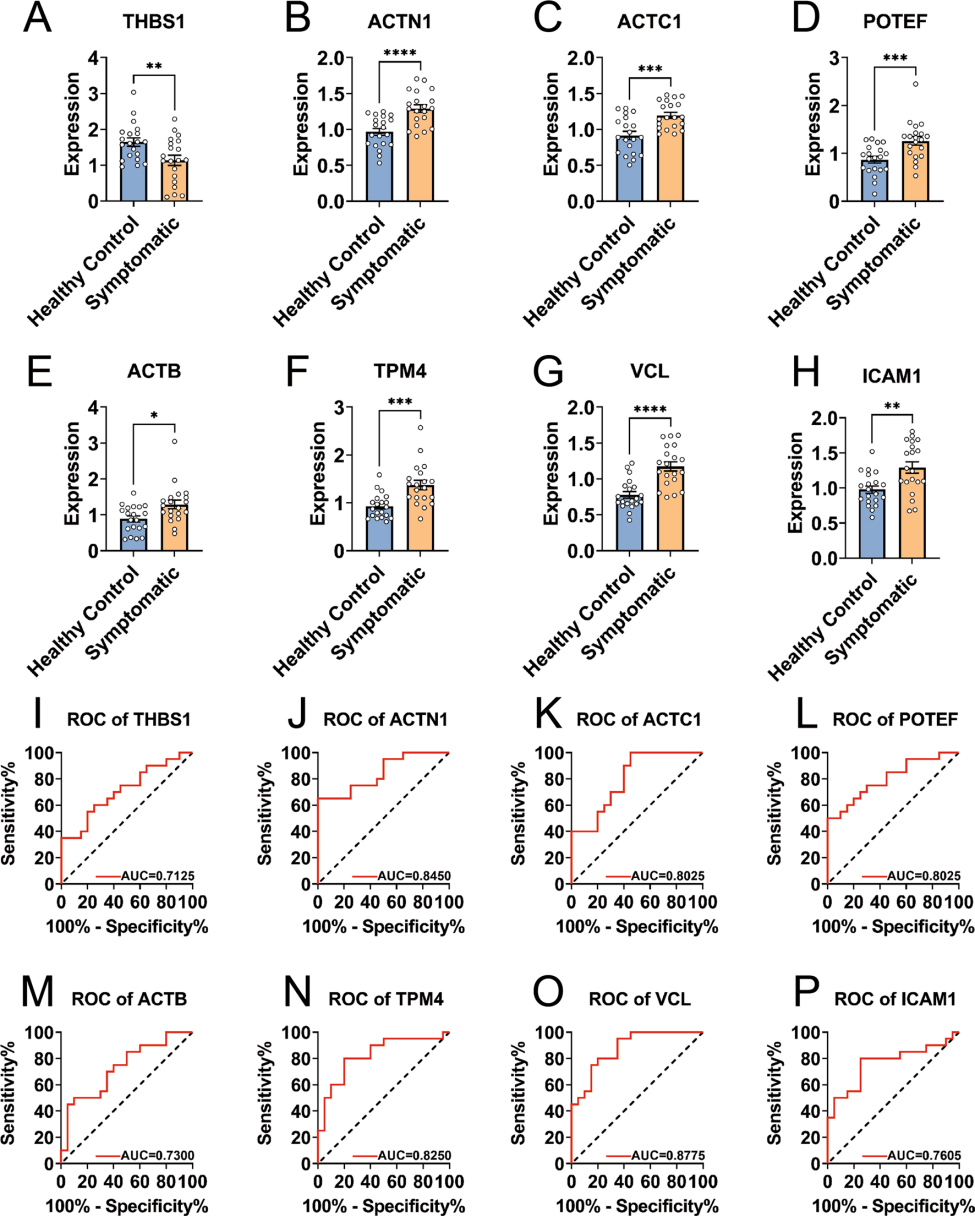
Validation of core protein expression in the validation cohort. **A**–**H** This series compares the expression levels of eight key proteins between Omicron-infected and healthy individuals, illustrating significant differences. **I**–**P** Diagnostic Receiver Operating Characteristic (ROC) curves for the same eight core proteins demonstrate their potential as biomarkers for distinguishing between Omicron and healthy samples. The Area Under the Curve (AUC) for all eight hub proteins exceeds 0.7, indicating a promising diagnostic capability. *P < 0.05. **P < 0.01.***P < 0.001. *ROC* receiver operating characteristic, *AUC* Area under Curve

The validation phase confirmed that the eight differentially expressed hub proteins significantly differed between the healthy control group and Omicron patients (P < 0.05) (Fig. 4A–H), also demonstrating diagnostic significance (Fig. 4I–P). Specifically, the area under the curve (AUC) for THBS1 was 0.7125 (95% CI 0.5527–0.8723), for ACTN1 it was 0.8450 (95% CI 0.7247–0.9653), for ACTC1 it was 0.8025 (95% CI 0.6672–0.9378), for POTEF 0.8025 (95% CI 0.6667–0.9383), for ACTB 0.7300 (95% CI 0.5733–0.8867), for TPM4 0.8250 (95% CI 0.6924–0.9576), for VCL 0.8775 (95% CI 0.7743–0.9807), and for ICAM1 0.7605 (95% CI 0.6113–0.9187).

### Omicron-associated metabolomic features and functional changes

**Fig. 5:**
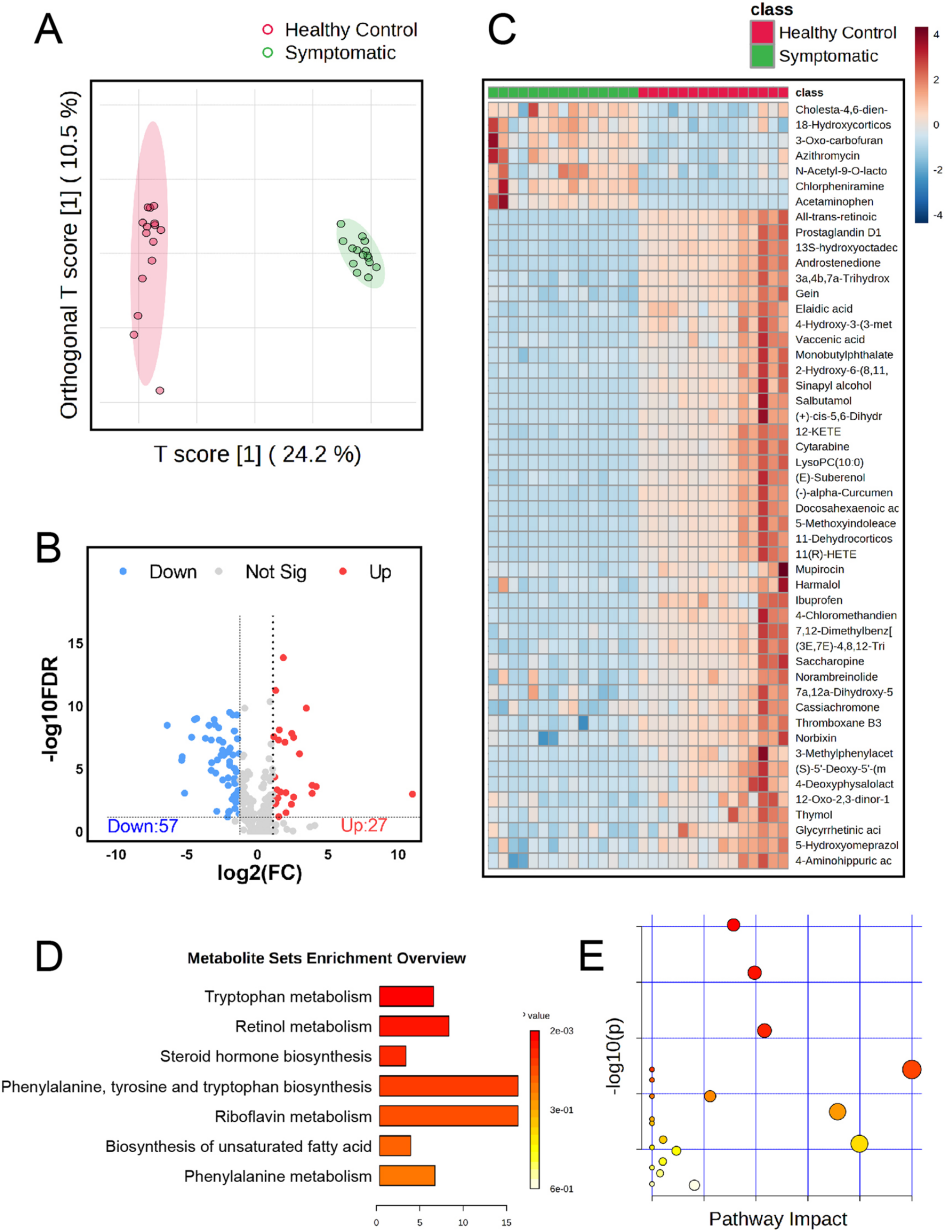
Metabolomics analysis. **A** Orthogonal Partial Least Squares-Discriminant Analysis (OPLS-DA) differentiates between healthy controls and Omicron-infected groups.; **B** Volcano plots reveal Differentially Expressed Metabolites (DEMs) between the two groups; **C** A heatmap illustrates the expression patterns of DEMs, highlighting significant variations.; **D**, **E** Gene Ontology (GO) and Kyoto Encyclopedia of Genes and Genomes (KEGG) analyses show that the identified metabolites are predominantly enriched in tryptophan metabolism, retinal metabolism, and steroid hormone biosynthesis pathways

Through the application of 4D non-targeted metabolomics techniques, we identified 146 differentially expressed metabolites (DEMs) out of 357 metabolites (Additional file 1: Table S4). Quality control was performed based on the distribution of the total metabolites by group. Orthogonal partial least squares-discriminant analysis (OPLS-DA) demonstrated a clear separation between the healthy control group and the Omicron group, indicating distinctive metabolomic profiles (Fig. 5A). A volcano plot revealed the expression differences among all metabolites, with 27 being upregulated and 57 downregulated (Fig. 5B). A heatmap displayed the top 50 differential metabolites between the two groups, underscoring the metabolic disparities (Fig. 5C). These differentially expressed metabolites were primarily enriched in pathways related to tryptophan metabolism, retinol metabolism, and steroid hormone biosynthesis, among others (Fig. 5D, E).

### Target drug prediction

**Fig. 6:**
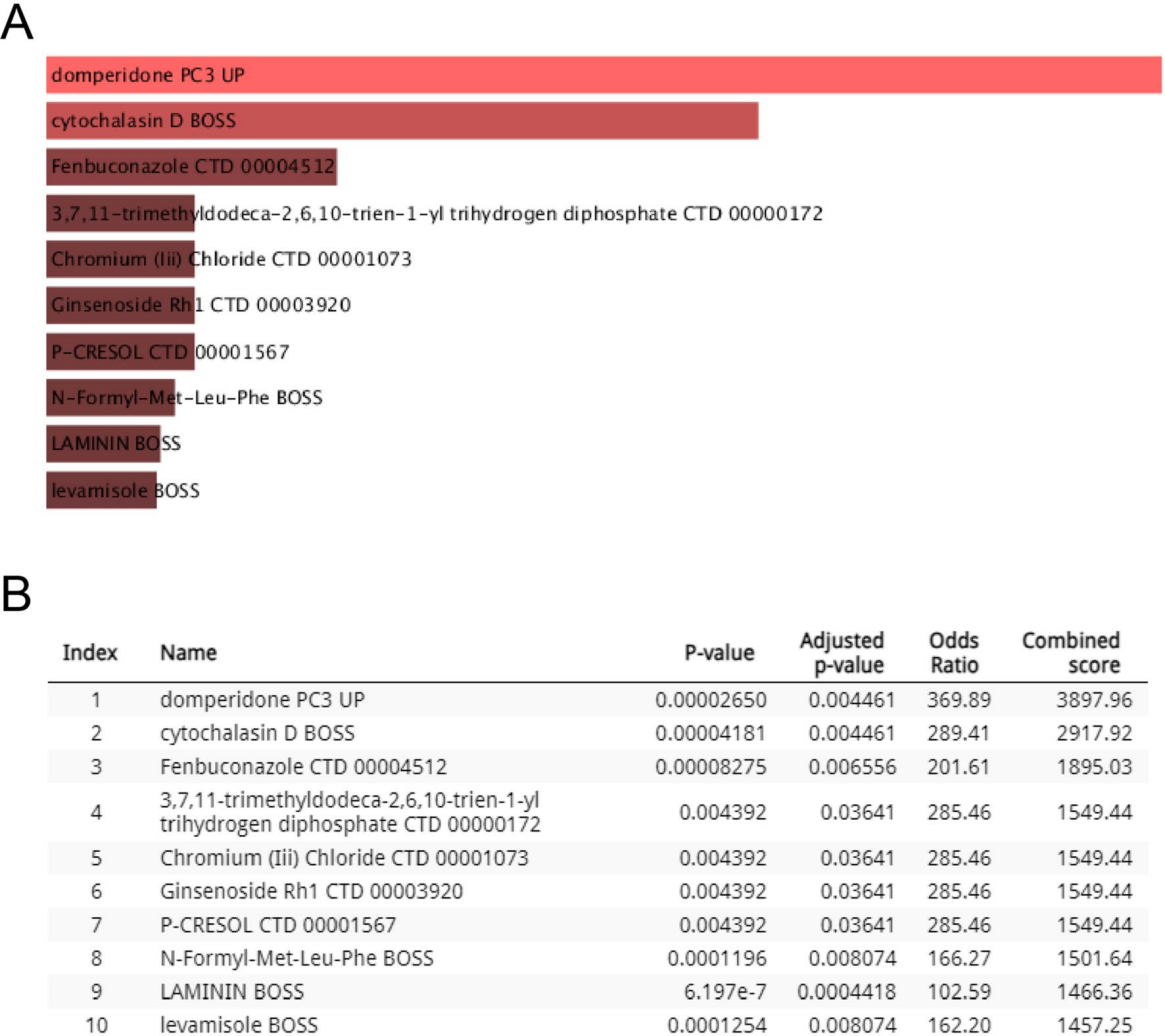
Top 10 Predicted Target Drugs. The top 10 predicted target drugs for combating Omicron infection, based on composite scores from DSigDB, are showcased. **A** Highlights the predicted target drugs that modulate the hub genes, with a bar graph illustrating their potential effectiveness. Notably, Domperidone and Cytochalasin D emerge as promising candidates for treating Omicron infection by targeting these eight critical proteins. **B** A table in DSigDB provides a detailed overview of these drugs and their associated scores, underlining their potential therapeutic value

The DSigDB database was used to predict potential target drugs associated with eight differentially expressed hub proteins that may treat Omicron infection by modulating the hub proteins. A total of 714 target drugs were finally predicted; the composite scores and corresponding target genes are listed in Additional file 1: Table S5. The top 10 predicted target drugs according to the composite scores are shown in Fig. 6.

## Discussion

Amid the current global health crisis, the rapid transmission of the Omicron variant has had profound impacts on economies and public health systems worldwide. Omicron, with its enhanced ability to evade the immune system, has shown significant transmissibility compared to previous SARS-CoV-2 strains [21]. Although our understanding of the host response dynamics to this new variant is limited, identifying biomarkers that can predict disease progression or assist in diagnosis is vital to meet the challenges posed by Omicron. Our study integrates clinical diagnostics with multi-omics analyses to compare biomarkers between individuals infected with Omicron and healthy subjects, identifying eight differentially expressed hub proteins—THBS1, ACTN1, ACTC1, POTEF, ACTB, TPM4, VCL, and ICAM1.

Significant mutations in the spike protein of Omicron enhance its ability to evade immune responses [22], which may be associated with severe clinical outcomes such as cerebral embolism, deep vein thrombosis, and respiratory failure [23]. These findings resonate with studies positioning antithrombin as a broad-spectrum inhibitor of coronavirus infection, suggesting that the coagulation pathway may be a key element in the course of Omicron infection [24]. However, the specific relationship between Omicron and the coagulation pathway has not yet been elucidated. Therefore, the relationship between the coagulation pathway and Omicron warrants deeper investigation. This study's analysis of coagulation markers, including Activated Partial Thromboplastin Time (APTT), Fibrinogen (FIB), and D-Dimer, in patients infected with the Omicron variant reveals significant alterations when compared to healthy individuals. These changes underscore a pronounced association with hemostasis, coagulation, and blood clotting pathways, mirroring the conclusions drawn in earlier research [25]. Further substantiating these findings, our GO and KEGG pathway analyses on eight central hub proteins identified a pronounced enrichment in biological pathways directly tied to hemostasis, coagulation, and blood clotting. This enrichment not only corroborates the observed changes in coagulation markers but also suggests that Omicron infection might precipitate distinct alterations in the biological processes integral to these pathways.

The interplay between inflammation and coagulation is well recognized, with inflammation able to trigger coagulation mechanisms, and vice versa [26]. In the context of COVID-19, the activation of coagulation and hemostasis pathways is considered to be mediated by the inflammatory system's tissue factors [27]. Even though Omicron exhibits biological characteristics different from previous variants, severe infections may still induce platelet activation and partial desensitization, thereby affecting the coagulation pathway [28], and the coagulation pathway plays a significant role in regulating pulmonary fibrosis, hemostatic disorders, and surfactant damage [29]. VCL, a protein located at the cytoplasmic face of cellular matrix and intercellular adhesions, is involved in linking integrins to the F-actin cytoskeleton and can act as a mechanical sensor transmitting forces to maintain cellular shape, serving as a dynamic regulator of cell adhesion [30, 31]. The high expression of VCL can enhance cellular adhesiveness, and its regulatory role in adhesion is crucial for biological processes such as leukocyte transmigration through epithelial or endothelial layers, thereby mediating the migration of leukocytes in the inflammatory response of COVID-19 [32]. ACTB protein, also known as β-actin, plays a vital role in various cellular processes such as cell division, migration, invasion, vesicle transport, and cellular structural regulation[33]. ACTB can modulate endothelial nitric oxide synthase activity, altering NO production and thus causing endothelial dysfunction, activating coagulation pathways and inflammatory responses [34]. THBS1 is a matrix glycoprotein released by activated platelets, involved in angiogenesis, inflammatory responses, platelet aggregation, cell apoptosis, and fibrosis [35]. A decline in THBS1 expression is associated with oxidative stress damage [36], and THBS1 is involved in the fibrin clot response to injury [37]. Furthermore, silencing of THBS1 can inhibit the activation of the NLRP3 inflammasome, reducing the levels of inflammatory cytokines, thereby reducing pneumonia caused by cytokine storms [38]. POTEF, as a member of the POTE ankyrin domain family, can regulate TLR signaling which is critical in innate immunity and is expressed in immune cells playing a significant role during cell invasion [39], and it can inhibit apoptosis and promote cell growth [40]. However, the role of POTEF in pneumonia caused by Omicron is not clear, and our findings suggest that POTEF is involved in tissue coagulation processes, participating in disease inflammation and cell regulatory responses.

ACTN1 is a cytoskeletal actin-binding protein [41] that, in addition to mediating sarcomere function, performs important non-muscle functions, such as the regulation of cytokinesis, cell adhesion, and migration [42]. Our study found that high expression of ACTN1 is associated with cell adhesion and is a significant factor in the development and progression of Omicron. ACTC1 is a cardiac-related protein expressed in regenerating muscle fibers in diseased mature skeletal muscle [43], and our study shows that in the Omicron group, ACTC1 expression is elevated through the actin filament organization pathway. TPM4 is a key actin-binding protein [44], and studies have shown that TPM4 expression is strongly increased in the later stages of megakaryocyte formation and that TPM4 has a functional role in thrombus formation in mammals [45, 46]. This is consistent with our findings, which indicate that TPM4 can act as a promoter in pathways such as focal adhesion, mediating the occurrence, development, and outcome of the disease. Studies suggest that focal adhesion is associated with mechanisms of cell migration [47], which can regulate mechanotransduction, cell migration, cell cycle progression, proliferation differentiation, growth, and repair [48], and our study finds focal adhesion to be related to the pathological process of the disease. Additionally, ICAM-1 is a cell surface glycoprotein and adhesion receptor that, besides being expressed on vascular endothelial cells, also functions on epithelial and immune cells, affecting inflammatory stimulation [49]. Studies indicate that ICAM-1 is associated with leukocyte-endothelial cell interactions and solute permeability changes [50]. Our study suggests that ICAM-1 has a significant association with the inflammatory exudation and endothelial barrier establishment caused by Omicron.

In addition, the tryptophan pathway can be affected by systemic inflammatory signaling factors, promoting immune suppression and evasion of immune surveillance in inflammation [51], thereby helping to suppress inflammation caused by Omicron. L-tryptophan is an essential amino acid that produces a variety of signal molecules through complex metabolic pathways [52, 53]. Studies also suggest that the L-tryptophan pathway can achieve immune suppression by depleting tryptophan, depriving proliferating T cells of essential amino acids [52, 53]. Indole-3-acetate (I3A), a metabolite depleted in the context of the microbiome and high-fat diets, has been proven to reduce the production of pro-inflammatory cytokines and inhibit cell migration towards chemokines, achieving the effect of alleviating inflammation [54]. These is consistent with our findings.

This study highlights the therapeutic potential of targeting eight differentially expressed proteins in treating Omicron infection, with Domperidone and Cytochalasin D being particularly noteworthy. Domperidone's antiviral properties, as demonstrated by stimulating prolactin secretion, suggest a dual role in enhancing both innate and adaptive immune responses [55], aligning with our findings and highlighting the necessity for further research on its clinical application parameters. Similarly, Cytochalasin D's inhibition of actin polymerization and its capacity to reverse PLT-induced suppression of cell apoptosis present a novel therapeutic pathway worth exploring [56].

The study acknowledges several limitations, such as the complexity of interpreting metabolomic data, which necessitates advanced bioinformatics and further biological validation. While eight potential biomarkers were identified, their clinical significance requires validation in a more diverse population. The absence of direct mechanistic evidence, focusing instead on associations, calls for further research to clarify how these changes affect disease progression. Despite these challenges, the study offers a comprehensive view of Omicron's pathophysiology, contributing valuable insights into coagulation and inflammation and underscoring the need for continued research to develop targeted clinical and public health strategies.

## Conclusion

In conclusion, our study highlights the significant impact of the Omicron variant on clinical and molecular levels. The identified hub proteins, enriched in coagulation and inflammatory pathways, underscore the intricate interplay between the inflammatory response and coagulation system in the context of Omicron infection. The biomarkers discovered offer a valuable resource for potential diagnostic and prognostic applications. While Omicron's unique biological characteristics necessitate further investigation, our findings contribute to the understanding of the pathophysiological mechanisms behind its clinical manifestations. However, the limitations of our study, include a call for further research with larger cohorts to validate these biomarkers and unravel the mechanisms of Omicron-induced pathology. Our work lays the groundwork for future studies aimed at developing targeted interventions to mitigate the effects of this challenging variant.

### Supplementary Information

**Additional file 1:** Table S1–Table S5.

## Data Availability

The data that support the findings of this study are available on request from the corresponding authors, upon reasonable request.

## Abbreviations

SARS-CoV-2: Severe acute respiratory syndrome coronavirus 2
VOC: Variant of concern
PLT: Platelets
WHO: The World Health Organization
ACE2: Angiotensin-converting enzyme 2
TMPRSS2: Transmembrane serine protease 2
COVID-19: Corona Virus Disease 2019
UHPLC: Ultra-high-performance liquid chromatography
UPLC: Ultra-performance liquid chromatography
DEPs: Differentially expressed proteins
DEMs: Differentially expressed metabolites
Log_2_FC: Log_2_FoldChange
GO: Gene ontology
KEGG: Kyoto encyclopedia of genes and genomes
PPI: Protein–protein interaction networks
OPLS-DA: Orthogonal partial least squares-discriminant analysis
VIP: Variable importance in projection
FDR: False discovery rate
IQR: Interquartile range
ROC: Receiver operating characteristic
AUC: The area under the curve
WBC: White blood cell
NEU: Neutrophil
LYM: Lymphocyte
MONO: Monocyte
EOS: Eosinophils
BASO: Basophil
PT: Prothrombin time
INR: International normalized ratio
PTTA: Prothrombin activity
FIB: Fibrinogen
APTT: Activated partial thromboplastin time
TT: Thrombin
DD: D-Dimer
CRP: C-reactive protein
SAA: Serumamyloid A
PCT: Procalcitonin
BP: Biological process
CC: Cellular component
MF: Molecular function
PCA: Principal component analysis
95% CI: 95% Confidence interval
I3A: Indole-3-acetate
THBS1: Thrombospondin 1
ACTN1: Alpha-actinin 1
ACTC1: Actin alpha cardiac muscle 1
POTEF: POTE ankyrin domain family member F
ACTB: Actin beta
TPM4: Tropomyosin 4
VCL: Vinculin
ICAM1: Intercellular adhesion molecule 1
PARK7: Parkinsonism associated deglycase
YWHAB: Tyrosine 3-Monooxygenase/Tryptophan 5-Monooxygenase Activation Protein Beta
